# When Does a Lot Become Too Much? A Q Methodological Investigation of UK Student Perceptions of Digital Addiction

**DOI:** 10.3390/ijerph182111149

**Published:** 2021-10-23

**Authors:** Luke Turner, Bridgette M. Bewick, Sarah Kent, Azaria Khyabani, Louise Bryant, Barbara Summers

**Affiliations:** 1 Leeds University Business School, University of Leeds, Leeds LS2 9JT, UK; luke.turner3141@gmail.com; 2 Leeds Institute for Teaching Excellence, University of Leeds, Leeds LS2 9JT, UK; 3 School of Medicine, University of Leeds, Leeds LS2 9JT, UK; l.d.bryant@leeds.ac.uk; 4 Division of Psychological and Social Medicine, University of Leeds, Leeds LS2 9JT, UK; sarah.kent@bdct.nhs.uk (S.K.); azaria_khyabani@hotmail.co.uk (A.K.); 5 Centre for Decision Research, Leeds University Business School, Leeds LS2 9JT, UK; bs@lubs.leeds.ac.uk

**Keywords:** Q method, digital addiction, student perceptions, internet addiction, problematic internet use

## Abstract

Despite the benefits of the internet and other digital technology, the online world has been associated with a negative impact on university student wellbeing. Many university students report symptoms of pathological internet use. Internationally, further research is needed to understand what student users of technology perceive to be problematic internet and/or digital use. The current study explores the range of perceptions that university students hold about ‘digital addiction’. We recruited 33 participants from a UK university into a Q-methodology study. Participants sorted, ranked, and commented on fifty-two statements representing the concourse of ‘things written or said about digital addiction’. The statements were identified from a comprehensive search of a wide variety of sources (e.g., newspapers, academic articles, blogs, and YouTube). Principal Component Analysis was used to identify four distinct viewpoints of ‘digital addiction’: (I) digital addiction is differentiated by the negative consequences experienced by addicted individuals; (II) digital addiction comes from our fascination with the online world; (III) digital addiction is an attempt to escape real world problems and impacts on mental health and relationships; (IV) digital addiction is defined by the amount of time we spend online. All four viewpoints share the perception that people do not realize they are digitally addicted because using and having digital devices on you at all times has become the social norm. There was also overall agreement that that those with ‘addictive personalities’ were more likely to be ‘digitally addicted’. Despite these similarities, complexity and contradictions within the viewpoints surrounding what digital addiction is and how it might be defined are apparent. The information found in this study provides important suggestions of how we might frame prevention and early intervention messages to engage students and ensure they develop the skills necessary to successfully manage their digital lives.

## 1. Introduction

Across the globe, overuse of the internet and other digital technologies have negatively impacted on productivity and wellbeing [1]. Digital devices have replaced other technologies (e.g., pen and paper), with digital reliance increasing as the online world becomes integrated into everyday life. When separated from their devices some individuals report experiencing anxiety related to separation from their device, with psychological and physical symptoms [2,3]; a powerful indication of the pervasive impact of digital devices.

In the UK, young people (16–34 years of age) are the group that spend the most time on digital devices [4]. The use of the digital world is particularly important for this age group, who have grown up using digital technology for learning, socializing, and communicating from a much younger age than older generations [5]. Despite the benefits of this enhanced digital technology, the online world is having a negative impact on university student wellbeing with many reporting symptoms of pathological internet use, e.g., [6,7].

The embedded nature of internet use as part of the student experience, for both academic and social purposes, has the potential to put students at risk of the negative consequences of problematic use of digital technology. Students are often reliant upon the digital world and mobile devices as the quickest, easiest and most convenient way to communicate and source information.

Problematic digital use is not currently a diagnosable condition with a unified definition. However, some have suggested that overuse can constitute an addictive behaviour [8]. Internet gaming disorder is recognized by the International Classification of Diseases (ICD-11 [9]) and is seen as worthy of more clinical research by the Diagnostic and Statistical Manual of Mental Disorders Fifth Edition (DSM-V [10]). Despite these advances, the terms ‘digital addiction’, ‘internet addiction, or ‘problematic internet use disorder’ have not yet been officially recognized in the Western World [11]. 

It is difficult to ascertain data for ‘problematic digital use’ or ‘internet addition’ in UK students with studies providing different estimates of likely prevalence (e.g., 3.2% [12] or 18.3% [13]). The differences across studies may be explained by differences in the standardized measures used, sample size, and timing of data collection. Currently, the majority of research investigating the personal and social factors involved in internet addiction are conducted in three Asian counties (Taiwan, China, and Korea) [5]; although the international evidence base is growing (e.g., Egypt [14], India [15], UK [12,13], and USA [16,17]). The field is dominated by cross-sectional studies using standardized measures of digital addiction to ascertain the prevalence of addiction and/or its relationship to other psychopathology. These studies explore internet/digital addiction as defined by the researcher; there is a paucity of evidence which explores the user’s perspective of the phenomenon of ‘problematic use’ of the online world and its relationship to user wellbeing. Internationally, further research is needed to understand what student users of technology perceive to be problematic internet and/or digital use.

Despite the lack of agreement around what constitutes problematic digital use and the likely size of the problem, there is a growing consensus that prevention and early intervention efforts are needed if the harms from problematic digital use are to be reduced. In particular, there are concerns around the impact of the digital world on social connectedness, productivity, sleep, dangerous driving, and mental health. Before we can successfully intervene, we must first understand the phenomenon.

There is a paucity of research investigating how people, including students, view digital addiction. Do people perceive digital addiction as just a part of the modern world we live in rather than a problematic addiction or dependency? Is digital addiction viewed as a serious issue that needs addressing? Understanding how students perceive and think about their own use of the online world and about digital addiction will enable the creation of prevention and intervention programs targeting behaviours and consequences that are consistent with how individuals understand and make sense of problematic use of the online world. Such interventions are more likely to resonate with individuals, and thereby increase levels of engagement and the ultimate success of future efforts to support people as they learn to effectively cope with the stresses and strains of the online world.

The current study used Q Methodology to explore the range of perceptions that university students hold about ‘digital addiction’ and the commonalities and differences between these perceptions.

## 2. Materials and Methods 

Ethical Approval for this project was given by Leeds University Business School AREA ETHICS committee application 11-004. 

### 2.1. Design

Cross-sectional online survey including online Q-sort and a short questionnaire detailing demographics and assessing the level of internet addiction (further details provided below). The online Q-sorting software POETQ was used to collect both quantitative and qualitative data [18]. 

### 2.2. Q methodology 

#### 2.2.1. Description of Q Methodology

Q Methodology is designed to identify the range of viewpoints held in relation to a topic of social interest within a given population who have a stake in how that topic is debated. It is a useful approach to study areas where tensions in the debate are known to exist—such as ‘digital addiction’ [19]. The method starts from the position that for each socially debated topic there is a ‘flow of communicability’, which is called the concourse [20]. The concourse consists of the things that are written or said about a topic that can be ‘socially contested, argued about and debated…. matters of values and beliefs’ [21]. The method uses a Q-sorting technique, where participants respond to a set of statements extracted from the concourse to indicate their agreement or disagreement with each statement. Factor analytic techniques are then used to identify where groups of Q sorts are similar or different to each other [22]. The pattern of statement placement for each factor is interpreted qualitatively and a narrative is created that represents a distinct point of view on the topic being investigated. 

#### 2.2.2. Sampling the Concourse and Creating the Q-Set for This Study

The concourse of this study was defined as ‘things written or said’ about digital addiction. In developing the Q-statements a wide variety of sources (*n* = 54) were consulted (e.g., newspapers, academic articles, blogs, and YouTube). The source information was read for examples of what people were thinking and saying about digital addiction. The views included in the captured material varied from digital addiction being seen as a mental health disorder through to the idea that digital addiction is not a problem and is just part of the modern world. As sources were discovered, viewpoints not already represented were added. As the number of sources read increased key concepts reoccurred until no new ideas were uncovered; at this point it was deemed saturation had been reached.

The viewpoints were grouped into overarching categories (e.g., causes, symptoms, consequences, treatment, and types of digital addiction). LT and BB then worked together to develop Q-statements that represented each distinct viewpoint. The aim was to create statements that represented each individual idea contained within each category; this stage resulted in 190 statements. The 190 statements were thematically analysed [23]. Fifty-two statements from the concourse were selected as representative of the full sample, and piloted by three University of Leeds students, BS, and LB. The pilot resulted in amendments to wording to enhance clarity of meaning and understanding. Following the amendments, the final Q-set consisting of 52 statements for use in the questionnaire was created.

### 2.3. Additional Data: Background Characteristics of the Sample and Assessment of Digital Addiction

Students were asked about their age, gender, affiliated School/Institute and level of study. Students completed the Internet Addiction Test (IAT; [24]). The 20-item IAT was modified to reflect the evolution in digital tools and devices that altered the way we access the internet (for example the development of ‘apps’ and ‘smartphone notifications’—e.g., ‘How often do you check your email before something else that you need to do?’ was modified to ‘How often do you check social media (e.g., Facebook, Messenger, WhatsApp, Snapchat, Viber), e-mail online and/or on your phone before doing something else that you need to do?)’. The scale measures the presence and severity of internet dependency. Total scores on the IAT vary from 0 to 100 with a higher score representing higher severity of dependence. Scores can be classified as normal internet usage (0 to 30), a mild level of internet addiction (31 to 49), a moderate level (50 to 79), and severe dependence on the internet (80 to 100). Students also reported how often they check their feeds (e.g., Facebook notifications, and Twitter alerts).

### 2.4. Study Population

In August 2016 students from the University of Leeds were invited to participate in the current study. Participants were approached by LT and invited to participate. LT approached students who were on campus (e.g., libraries, refectory, and university union). In addition, the Q-sort invitation was advertised to students via their departments. In total 52 students were invited to participate. Thirty-three students completed an online Q-sort. Mean age was 21.85 years of age. Approximately two-thirds (*n* = 23) identified as female with the remaining participants (*n* = 10) identifying as male. The majority of participants were undergraduate (*n* = 23 undergraduate, *n* = 6 postgraduate, and *n* = 4 PhD students). The average modified Internet Addiction Test score was 34.21 (*n* = 14 normal, *n* = 16 mild, *n* = 3 moderate, and *n* = 0 severe). Table 1 summarizes the sample characteristics.

### 2.5. Incentive

Students who completed the registration survey could opt into a prize draw to win a first prize of GBP 10, with second and third prizes of GBP 5. Each participant who completed the Q-sort received GBP 5. 

### 2.6. Data Collection and Analysis

The Q-sort data (quantitative) were analysed using PQMethod version 2.33 (Schmolck, Munich, Germany) [18]. Interpretation of the factors used statistical outputs from PQMethod alongside qualitative data collected as part of the online procedure. 

**Figure 1:**
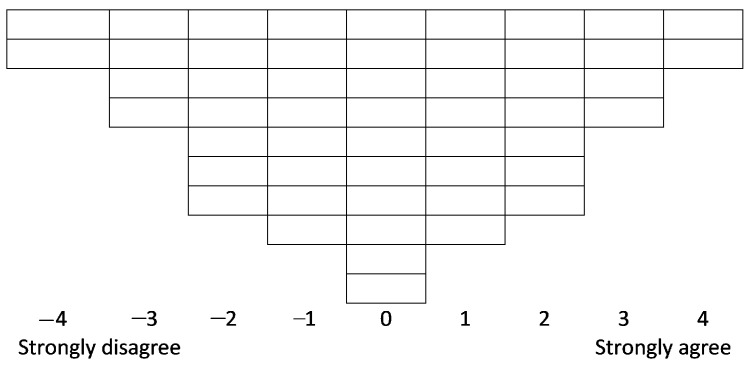
The Q-sorting grid.

Through a series of iterative steps, the POETQ online platform [18] enabled participants to systemically rank the 52 statements according to agreement or disagreement, ultimately placing the items into cells on a normally distributed grid (see Figure 1; [25,26]). Qualitative data were also collected within POETQ: participants were asked to complete free text entries to give their reasons for selecting the statements they ranked highest and lowest in terms of agreement or disagreement. The Q-sort data were downloaded into an Excel data file. Quantitative data were submitted to statistical analysis using PQ-Method [27] a program tailored to the requirements of Q methodology. Qualitative data entered within POETQ were extracted from the Excel file and used to support interpretation of the factors.

### 2.7. Factor Extraction and Interpretation

Using PQMethod, factors were identified using Principal Component Analysis (PCA) and Varimax rotation. Established strategies were employed to identify the maximum number of interpretable and distinct viewpoints to extract and take forward for interpretation [26,27]. Firstly, factors with an eigenvalue greater than one (Kaiser–Guttman criterion) with at least two significantly loading Q-sorts were plotted on a simple line graph (scree plot); factors falling around the point where the line changed slope and before the point where the line levelled off were considered for rotation. Secondly, Humphry’s rule was applied using the cross-product of the two highest loading sorts on each factor [26]. The Q Methodology software uses a weighted formula to merge the exemplars to create an average score for each of the 52 statements. This is called the factor array and represents an idealized Q-sort for each viewpoint [26].

A four-factor solution was identified as most suitable for interpretation: these four factors accounted for 51% of the variance; 18 of the 33 students were mapped significantly into one of the factors, each factor had a least two significantly (*p* < 0.01) loading exemplar Q sorts. The factor arrays are shown in Table 2.

Established methods of Q factor interpretation were applied to the factor solutions. Interpretation requires careful synthesis of the quantitative and qualitative data collected during the Q-sorting activity. The information produced by PQMethod is used to inform the first level of interpretation. Using the factor arrays, the highest and lowest scores assigned to particular statements for each factor are considered first, along with the statements that distinguish between any one factor and all other factors at *p* < 0.01. A deeper level of interpretation then follows whereby the idealized Q-sort is considered as a whole alongside the qualitative information provided by the participants in this study collected via the online platform. The qualitative data is used as a ‘validity check’ against the researchers’ interpretation and to throw more light on the importance of certain statements to this particular viewpoint and the meaning they may have.

## 3. Results

The results are presented as a set of narrative descriptions of the different viewpoints identified via the factor interpretation. Qualitative data from participants who were exemplars of each factor are used to illustrate and provide evidence to support our interpretation.

### 3.1. Viewpoint I: Digital Addiction Is Differentiated by the Negative Consequences Experienced by Addicted Individuals

Viewpoint I was exemplified by four exemplars. The four exemplars have an age range of 21–25 years and three of them are female. Their IAT scores ranged from 23–56 (one normal, two mild, and one moderate).

While engaging with the digital world is now the norm and inevitable, addiction is not inevitable and there is not a strong view that the online world is inherently addictive. Each individual can decide whether or not they will ‘give-in’ to the excitement of the online world and can control whether or not they ‘become’ addicted. Conforming to social pressures to be online does not lead to digital addiction.

In this viewpoint, digital addiction is a problem or disorder distinct from general high-usage and addicts will need professional support to overcome it. The sense of anticipation and the rewards of ‘waiting for the next text or status update’ or experiencing a buzz through ‘getting likes’ or winning an online game may cause digital addiction. In addition, people get addicted to being online because social media allows them to portray ‘perfect lives’, controlling their image.

*‘Using digital devices and social media has become a part of individuals’ everyday lives, I think it would be unrealistic to try and deter people away from using these platforms… encouraging people to take time away from these platforms reminds people that they can function without them and it isn’t a necessity.’* (Female, PG, age 22 years, mild IAT)

’Digital addiction’ can be defined by consequences and harm that occur for the individual in the physical-world, such as use of digital devices ‘getting in the way’ of essential life activities like eating and sleeping, engaging in risky behaviour (texting while driving), and experiencing substantial financial difficulties, e.g., job loss or debt through buying games; loss of relationships due to prioritizing the online world over real-world interaction is also a sign of digital addiction.

*‘It is not okay to be digitally addicted, and I think the issue is, is that most people aren’t aware that they have a ‘problem’ and that it needs to be ‘fixed”* (Female, UG, age 21 years, moderate IAT)

*‘This is a serious negative and life threatening impact. Anything which has this effect should be viewed as an addiction’* (Male, UG, age 21, mild IAT)

### 3.2. Viewpoint II: Digital Addiction Comes from Our Fascination with the Digital World

Viewpoint II was exemplified by nine exemplars. The nine exemplars have an age range of 18–28 years and seven of them are female. Their IAT scores ranged from 24–52 (three normal, five mild, and one moderate).

It is believed in this viewpoint that digital addiction is caused by fascination with the online world and a compulsion to explore it. The online world is designed to be addictive and so the fact that some people become addicted to it is an expected product of the digital environment and the amount of digital content and activities available.

*‘Everything is designed to take time and keep you coming back. Games in particular are guilty of this, for example candy crush refreshing ‘lives’ over a period of time… Obviously the internet is huge, there is something for everyone and it is so easily accessible’* (Male, UG, 18 years, IAT normal)

Digital addiction is not just a problem in some countries, such as China, Taiwan, and Korea. In this view it is not associated with negatively viewed characteristics, such as low self-esteem, mental illness, or obesity. Digital addiction is not gendered.

*‘Just because an individual is obese doesn’t mean they’re going to be more likely to be digitally addicted… they may spend a lot of time online but that doesn’t mean they’re addicted… all genders can experience the same things online… both as likely to become digitally addicted…’* (Female, UG, age 19 years, IAT mild)

You cannot define digital addiction by the amount of time spent online, being a compulsive user, or getting absorbed in the online world and losing track of time. Like other viewpoints, Viewpoint II believes that people do not realize that they are digitally addicted because having digital devices on you at all times is now the social norm.

*‘Phones/laptops/tablets are now an extension of our lives offline. We use them to communicate, to find and locate things, to play, to search for information, to read, to book appointments, etc.’* (Female, PG, age 23 years, IAT mild)

### 3.3. Viewpoint III: Digital Addiction Is an Attempt to Escape Real World Problems and Impacts Mental Health and Relationships

Viewpoint III was exemplified by two exemplars. The two exemplars were 22 and 33 years. Both are female and have IAT scores in the mild range (35 and 37). 

In this viewpoint, digital addiction is a problem. It is not primarily defined by compulsive use of the online world or by quantities of time spent there but instead by the extent to which relationships in the physical world suffer. Other ‘addictive’ signs are impacts on eating and sleeping and financial difficulties. There is strong agreement also that digital addiction, like other addictions has a negative impact on mental health

*‘… relationships do suffer as a result of digital addiction… if addiction is significantly impacting on mental wellbeing and relationships then it is a problem that needs to be looked at… addiction has a negative impact on mental wellbeing irrespective of type’* (female, PhD, age 33 years, IAT mild)

Addicts may need professional help as they cannot treat themselves. In contrast to viewpoint II the online world is not seen as inherently addictive but addicts may use the online world to escape from real life problems. 

Personal characteristics such as age, gender, obesity, or previous mental health problems are not seen as particularly related to digital addiction.

*‘Strongly disagree that females are more likely than males to become digitally addicted… would like to think both are equally likely to be addicted…’* (female, PhD, age 33 years, IAT mild)

### 3.4. Viewpoint IV: Digital Addiction Is Defined by the Amount of Time One Spends Online

Viewpoint IV was exemplified by three exemplars. The three exemplars have an age range of 19–21 years and two of them are female. Their IAT scores ranged from 26–51 (two normal and one mild).

In this viewpoint digital addiction is about the (excessive) amount of time spent online. Being a compulsive user of the internet does not necessarily mean you are addicted. While content in the online world is designed to be ‘addictive’, digital addiction is not necessarily a disorder that warrants professional help, but it does have a negative impact on mental health and needs to be addressed. Spending less time online and regularly having a ‘detox’ from the online world is needed. Addiction is not a consequence of the digital world allowing people a hidden place where they can do what they want, nor is it a consequence of the ‘buzz’ from being online.

*‘Everyone now days is on their phone or iPad or laptop… there is no need for digital addiction. Everyone just needs other hobbies… someone who is digitally addicted can just change their lifestyle and should limit the time spent on the internet… it is a problem because it affects people’s actual physical lives’* (Female, UG, age 20 years, IAT normal)

*‘… The majority of people use the internet a lot and are not addicted. Internet usage is inevitable for many occupations so many people are compulsive users. Addiction is not inevitable just through usage. There’s nothing inherently and universally addictive about the internet’* (Male, UG, 21 years, IAT normal)

### 3.5. Consensus Statements

Nine consensus statements, i.e., those that did not distinguish statistically between any pair of factors were found (see Table 2; consensus statements are shaded in grey). 

There was a shared perception that consistent regular engagement with the online world is now the social norm and that this can mean people do not realise when they are digitally addicted. Getting aggressive on losing access to digital devices had mild level of agreement across factors as a sign of addiction, but lack of attention to the physical world when looking at devices did not, e.g., when crossing a road. There was general (but not strong) agreement that those with addictive personalities were most at risk, but no strong perception either way that those more or less likely to be digitally addicted are socially awkward and more comfortable talking to people online than in the physical world. 

There was disagreement with the suggestion that digital addiction was a problem primarily because of physical consequences such as backaches (statement 26). Instead, substantial consequences such as job loss or other financial negative implications were more important markers of addiction.

## 4. Discussion

This is the first study to systematically gain an understanding of how university students think and feel about what is being written and said about the phenomenon of ‘digital addiction’. Four distinct viewpoints were found in this study. All viewpoints share the perception that people do not realize they are digitally addicted because using and having digital devices on you at all times has become the social norm. This suggests that the majority of students are unlikely to proactively engage with interventions that are overtly marketed as being for students who self-identify as being digitally addicted. Marketing the ability for interventions to reduce emotional distress, reduce loneliness and improve behaviours that students are motivated to change is more likely to increase the perceived relevance and subsequent engagement of students in interventions. There was also overall agreement that that those with ‘addictive personalities’ were more likely to be ‘digitally addicted’. Despite these similarities, complexity and contradictions within the viewpoints surrounding what digital addiction is and how it might be defined are apparent. The information found in this study provides important suggestions of how we might frame prevention and early intervention messages to engage students and ensure they develop the skills necessary to successfully manage their digital lives.

The general pervasiveness of the online world plays a role in all four viewpoints. There is mixed evidence of effectiveness of ‘detoxing’ from the digital world by taking a period of abstinence [28] and the findings from the current study suggest that taking a ‘digital detox’ for any length of time is unlikely to be palatable for the majority of students. In particular, the majority of UK universities now blend online and face-to-face throughout degree programs [29]. Students are therefore encouraged to engage regularly with online spaces. Popular mobile instance messaging [30] and social media applications [31] are promoted as ways for students to connect with fellow students and to stay updated on extracurricular activities. Given this context it is unlikely that abstaining from the online world will be a viable option for many university students; therefore, prevention and early intervention efforts might do well to find other ways to interrupt problematic habitual behaviours. 

Viewpoints differ in the extent to which digital addiction is perceived to be due to individual characteristics, the design of the digital world, or one’s own emotional reaction to engagement with the digital world. Knowledge of the range of mechanisms believed to explain why and how individuals become digitally addicted provides insight into how we might begin to frame prevention and early intervention efforts. In particular, the current results suggest that public health prevention social marketing messages that raise awareness of the multiplicity of likely underlying causes (e.g., persuasive technology [32]) and consequences (e.g., loneliness [33]) that resonate with students have a greater chance of engaging the student population than campaigns that focus on changing digital behaviour per se as a single motivator for change. 

Whether or not time spent online helps to define if someone is digitally addicted differed across the viewpoints, as did the importance, and range of, consequences that defined whether someone was deemed to be digitally addicted. For some students, emotional consequences and impact on physical-world relationships were key to defining someone as digitally addicted. This suggests that some students might be receptive to interventions that build on this awareness and provide tools for early detection of problematic habits and behaviours. There was a general consensus that consequences for one’s mental health and wellbeing were of greater concern that those to physical health. Interventions that help students to cope with the emotional stress and felt anxiety of dealing with the digital world are likely to be more appealing for many students and address problems that resonate with their own perspectives. 

Viewpoints I, III, and IV strongly feel that it is not ok to be digitally addicted; it needs to be fixed, Viewpoint II also supports this, but to a lesser extent. Most groups think that digital addiction is an addiction in itself and not an addiction to an activity that has been taken online, although Viewpoint II is neutral. If this is the case, then it is unlikely that student support groups for things like gambling are likely to address the needs of the majority of students who are seeking help to modify their use of the online world.

Only Viewpoint IV expresses the view that that digital addiction is something that might be treated without the need for professional help; others believe that, once digitally addicted, people need the support of professionals to overcome the ‘addiction’. This suggests that if an effective way of getting students to acknowledge their addiction to the online world can be found then many students are open to being offered support. These findings also suggest that some students may be more willing to engage with self-help materials than traditional face-to-face interventions. Self-help could potentially include group support, (similar to alcoholics or gamblers anonymous in other contexts), although self-help in general would need careful pitching in that it is not clear whether addicts would wish to avoid the addiction label. 

Viewpoint IV gave some support to the idea that digital addiction might cover up some disorders such as social anxiety, although others did not agree. Viewpoints I and IV gave some support to the idea that those who are isolated might use the internet to make a virtual connection. Supporting students in adjustment to life where they are often away from family and friends for the first time might therefore help in reducing vulnerability to becoming addicted. There are promising indications that face-to-face group interventions that capitalize on the therapeutic benefits of Cognitive Behavioural Therapy and mindfulness can be effective in modifying smartphone addiction [34]. Should a group-based approach prove effective in addressing problematic internet use, the ability to connect with other students while in a therapeutic setting is likely to be a useful format for those students currently relying on the online world to build meaningful connections during their transition to university.

## 5. Study Strengths and Limitation

By Q-sorting, the participants are largely in control of defining what is relevant when defining digital addiction [26]. It is a strength that the concourse for this study was developed by and pilot tested with university students. This pilot testing increased the relevance of statements for students by enabling tailoring of language and grammar for this specific population. The study population includes representation of undergraduate and postgraduate students thereby increasing the likelihood that the findings will be of relevance to the wider student body. The proportion of participants in the current study who scored above the IAT’s range for normal internet use is high relative to estimates provided by other UK studies [12,13]. Students scoring within the normal range were included in the sample and therefore it is anticipated that their viewpoints are represented. The sample included no students classified as being severely impaired by their internet use. Further research is required to ascertain the generalizability of findings to non-student and to severely impaired student populations.

One limitation of the study is that it was only carried out at one University in the UK, but it provides a point of comparison for future work. Given point estimates for prevalence of digital addiction differ across geographical locations it is likely that there will also be cultural norms and perceptions around digital addiction; further research is needed to empirically test this hypothesis. This Q-methodology study has identified four discrete viewpoints within the shared UK university student discursive space; the study is unable to comment on viewpoints that might be present in other spaces (e.g., Chinese universities). This study is unable to comment on how student’s perceptions change across time; longitudinal research is needed to understand if perceptions of the phenomenon of digital addiction remain static over time. 

Despite these limitations, the results from the current study serve as an important step in understanding the convergence and in understanding differences in perceptions of digital addiction among UK university students. 

## 6. Conclusions

This study gives us insights into the views of UK university students on digital addiction. Most viewpoints in this study see digital addiction as a problem, although recognizing that the online involvement is pervasive, and is widespread in everyday life. The different understandings of why digital addiction arises can be helpful in designing acceptable interventions. Most viewpoints recognized a need for professional help to overcome addiction and get away from its harmful effects.

## Figures and Tables

**Table 1:** Characteristics of the study participants.

	*N*	(%)
Students	33	
Age Mean (SD)	21.85	(3.51)
Gender		
Female	23	(70)
Male	10	(30)
Current student status		
Undergraduate	23	(70)
Postgraduate	6	(18)
PhD	4	(12)
Faculty		
Arts, humanities, and cultures	11	(30)
Biological sciences	2	(5)
Business	6	(16)
Engineering and physical sciences	7	(19)
Environment	4	(11)
Medicine and health	5	(14)
Social sciences	2	(5)
Internet Addiction Test categorisation		
Normal internet use	14	(42)
Mild impairment	16	(48)
Moderate impairment	3	(9)
Severe impairment	0	(0)
Frequency of checking feeds		
Every 5 min	3	(9)
Every 15 min	2	(6)
Every 30 min	9	(27)
Once an hour	7	(21)
5–6 times a day	10	(30)
3–4 times a day	1	(3)
2 times a day	1	(3)

**Table 2:** Factor arrays: Scores against each item by viewpoints (greyed out statements indicate statistical consensus).

No.	Statement	F1	F2	F3	F4
1	Spending excessive amounts of time on digital devices means you are a digital addict.	0	0	−3 *	3 *
2	Being digitally addicted has a negative impact on your mental well-being.	2	0 *	4	4
3	Digital addiction is caused by the sense of anticipation. Waiting for the next text, status update or reward hooks us in.	3	2	1	0
4	The online world is addictive.	1 *	4	−2	4
5	Putting yourself at risk by looking at your digital device and not paying attention to the real world (e.g., texting when driving, tweeting while crossing the road) means you are a digital addict.	4 *	−2	−1	−2
6	Digital addiction is most problematic in China, Korea and Taiwan—digital addiction is not an issue in the United Kingdom.	−2	−4	−3	−4
7	Someone who is socially awkward is more likely to become digitally addicted because online platforms (such as fantasy games) makes them feel confident.	0	1	0	0
8	Feeling depressed because you can’t get online means you are a digital addict.	2	−2 *	2	1
9	People become digital addicts because the digital world allows them a private hidden place where they can do what they want.	0	0	0	-3 *
10	If you are digitally addicted you don’t need professional help. Digital addicts can treat themselves.	−3	−2	−2	2
11	Digital addiction is negatively affecting students’ academic life because they are choosing to go on their digital device rather than study.	−1	−2	2	2
12	Regardless of your age, if you engage with the online world you have the same chance of being digitally addicted.	−1	−1	2 *	−2
13	It is okay to be digitally addicted. It is not a problem that needs to be fixed.	−4	−1 *	−4	−4
14	If you find yourself often losing track of time due to using your digital device you are a digital addict.	−3	−3	−1	0
15	If you use the digital world to escape from your real life problems you are digitally addicted.	0	−1	2	0
16	Digital addiction is a consequence of the endless supply of information and things to do online.	0	3 *	−1	1
17	If you lie or hide the amount of time you use your digital device then you are a digital addict.	−1	−1	1	−1
18	Being unable to feel like you were at an event unless you posted it online is a sign of being digitally addicted (i.e., if you didn’t post about it you feel like you weren’t there).	1	−1	1	−1
19	You are more likely to be a digital addict if you already have an addictive personality.	1	2	2	2
20	People who are isolated in the real world and who lack real world relationships become digitally addicted to recreate virtual relationships online.	2	0	0	1
21	The main reason people are addicted to the online world is because of the buzz you get (e.g., feel good when you receive likes, the good feeling from knowing you’re up to date on social media, the rush when you win an online game).	2	1	0	−3 *
22	Someone who needs to keep checking digital devices for fear of missing out is digitally addicted.	1	−1	1	0
23	To prevent becoming digitally addicted people should regularly have a digital detox (i.e., go offline for a while).	1	−1	0	3 *
24	People who are digitally addicted are those with low self-esteem and feelings of inadequacy.	0	−3 *	0	−1
25	If you quit digital use and you experience anxiety then you are a digital addict.	2	1	2	−2 *
26	The main reason digital addiction is a problem is because of the physical consequences (e.g., backaches, weight gain, headaches, short sightedness).	−2	−2	−2	−1
27	Given all the benefits we gain from the online world it is a necessary evil that some people get digitally addicted.	−1	2	1	−2
28	Digitally addicted people feel more comfortable talking to people online than they do talking to people in the real world.	0	0	0	1
29	Being digitally addicted means that real life relationships will suffer because digital devices take priority over the people in front of them.	4	0	4	0
30	You can’t define digital addiction by the amount of time spent online - what one considers addictive use depends on the person.	−2	3	3	−3
31	People become digitally addicted as a result of their desire to increase their online popularity (e.g., number of relationships they have online).	1	1	0	−2
32	The digital world is a medium, peoples true addiction is what they seek online (e.g., Gambling, gaming, pornography). You can’t be addicted to the medium.	−1	0	−2	−1
33	Females are more likely than males to become digitally addicted.	−2	−3	−4	−1
34	Looking at your digital device and not paying attention to the real world (e.g., walking down the street) means you are a digital addict.	−1	−2	−1	−1
35	Digital addiction is inevitable because digital devises are essential for daily tasks (e.g., banking, looking up medical information, booking appointments).	−4 *	1	1	0
36	If you feel aggressive when access to the digital world is taken away from you (e.g., losing signal, battery dies) then you are digitally addicted.	1	1	1	0
37	People with mental health problems are more likely to be digitally addicted.	−1	−3	−3	1
38	People become digitally addicted because the digital world (e.g., apps, games, content) is designed to be addictive.	1	1	−1	2
39	People don’t realise they are digitally addicted because using and having digital devices on you at all times is the social norm.	3	3	3	3
40	Being digitally addicted is not a disorder but is covering up other disorders - such as social anxiety or panic disorder.	−3	−1	−2	1
41	People get digitally addicted because social media allows them to portray the perfect life online.	3	2	0	1
42	People who have jobs that require them to use the digital world are more likely become digitally addicted even outside of work.	−2	0	0	−2
43	Digital addicts like to stay in contact with their digital device because it makes them feel they are more connected to the world.	0	2	1	0
44	Digital addiction (e.g., an addiction to social media) is making people more egocentric and self-centred.	2	0	−2	2
45	Digital addiction is because digital technology fascinates us. We are compelled to digitally explore and find out more.	−1	3	−1	2
46	If you are obese you are more likely to be digitally addicted.	−2	−4 *	−2	0 *
47	If using your digital device has caused a substantial negative impact on your economic situation (e.g., job loss, in-game purchases) you are digitally addicted.	2	2	2	1
48	A sign of a digital addiction is someone whose use of digital devices gets in the way of essential life activities (e.g., eating and sleeping).	3	2	3	2
49	Needing to always have backup digital devices and/or charging devices with you in case the battery dies means you are a digital addict.	−2	−2	−3	−3
50	Being a compulsive user of the internet does not necessarily mean you are digitally addicted	0	4	−1	3
51	Over time someone who is digitally addicted will need to spend longer online to get the same amount of satisfaction.	0	0	1	−2 *
52	Conforming to social pressure to always be online leads to digital addiction.	−3	1 *	−1	−1

* Indicates a statement where the factor level score distinguishes from all other factors at *p* < 0.01. Grey shading indicates consensus statements.

## Data Availability

Conditions of ethical approval precludes data sharing. Data is not available to be shared.
